# Hepatic Portal Venous Gas: An Ill Omen or a Misleading Sign

**DOI:** 10.1055/s-0040-1702919

**Published:** 2020-03-03

**Authors:** Shruti Mehta, Gunjan S. Desai, Saumil Shah, Hitesh Mehta, Aniruddha Phadke

**Affiliations:** 1 Department of Internal Medicine, Lilavati Hospital and Research Centre, Mumbai, Maharashtra, India; 2 Department of Surgical Gastroenterology, Lilavati Hospital and Research Centre, Mumbai, Maharashtra, India; 3 Department of Internal Medicine and Medical Gastroenterology, Lilavati Hospital and Research Centre, Mumbai, Maharashtra, India

**Keywords:** necrotizing enterocolitis, bowel gangrene, leukemia

## Abstract

Hepatic portal venous gas (HPVG), a rare radiological finding, is historically considered an ominous sign with 100% mortality rates. The dictum that HPVG warrants surgical intervention is challenged in the recent literature. This is because of the identification of various causes of HVPG other than bowel gangrene. Most of these newly identified causes can be managed conservatively. However, bowel gangrene, if missed, is fatal. Hence, sound clinical judgment and accurate diagnosis based on specific clinical parameters and imaging findings are important. We present a case of a young male with tumor lysis syndrome and neutropenic sepsis. He underwent treatment for a relapse of T-cell acute lymphocytic leukemia and presented with abdominal pain and distension. Computed tomography (CT) scan showed HPVG, and the differential diagnosis was neutropenic colitis or pseudomembranous colitis, with steroid use as the probable cause. The patient was managed conservatively. The case emphasizes that the evaluation for a specific cause of HPVG is important to reduce unnecessary surgery. A succinct literature review provides the reasons for the changing mortality rates.

## Introduction

Hepatic portal venous gas (HPVG) is a rare radiological finding. It is defined as a branching radiolucency on radiograph (X-ray) or computed tomography (CT) scan within 2 cm beneath the liver capsule. 1 It was first described by Wolfe and Evans in 1955 in six infants with necrotizing enterocolitis. Subsequently, it was reported in 1960 in five adults with bowel gangrene associated with 100% mortality. 2 3 Early reports indicated that HPVG was diagnostic of bowel gangrene and that the presence of HPVG mandated laparotomy. However, the diagnosis of HPVG was based on X-ray of the abdomen. In the last two decades, with increasing use of CT scans, HPVG is being increasingly described due to other causes. Most of these cases can be managed conservatively. Recent studies demonstrate a decrease in mortality to 25 to 39%. 3 4 5 6

We report a patient with HPVG managed conservatively. We emphasize that HPVG is not an ominous sign by itself. It should always be evaluated, keeping in mind the entire clinical scenario to avoid negative laparotomy.

## Case Presentation

A 23-year-old gentleman with T-cell acute lymphocytic leukemia (ALL) was managed with rituximab, cyclophosphamide, doxorubicin, vincristine, and prednisone induction, and maintenance therapy. He had a relapse after one year of diagnosis and was started on prednisolone and rituximab salvage therapy. He presented with breathlessness and fever after the first salvage therapy cycle. Investigations revealed a total leukocyte count of 1,54,000/mm ^3^ (4,000–10,000), neutrophil (4%), serum calcium of 8 mg/dL (8.5–10.5), uric acid of 17.58 mg/dL (1.5–8), phosphorus of 5.8 mg/dL (3–4.5), lactate dehydrogenase of 4876 U/L (160–450), and creatinine of 1.66 mg/dL (0.5–0.9). The features were suggestive of tumor lysis syndrome, acute renal failure, and neutropenia. Peripherally inserted central catheter (PICC) was placed.

The patient developed fever due to a cubital fossa abscess around PICC. His left ventricular ejection fraction dropped to 20%. The abscess was drained and antibiotics were started. Five days later, he complained of abdominal distension and pain with nausea, diarrhea, and persistent fever. Examination revealed tachycardia and tenderness in the left iliac fossa. There was no abdominal free fluid and no guarding or rigidity on abdominal examination.

**Fig. 1:**
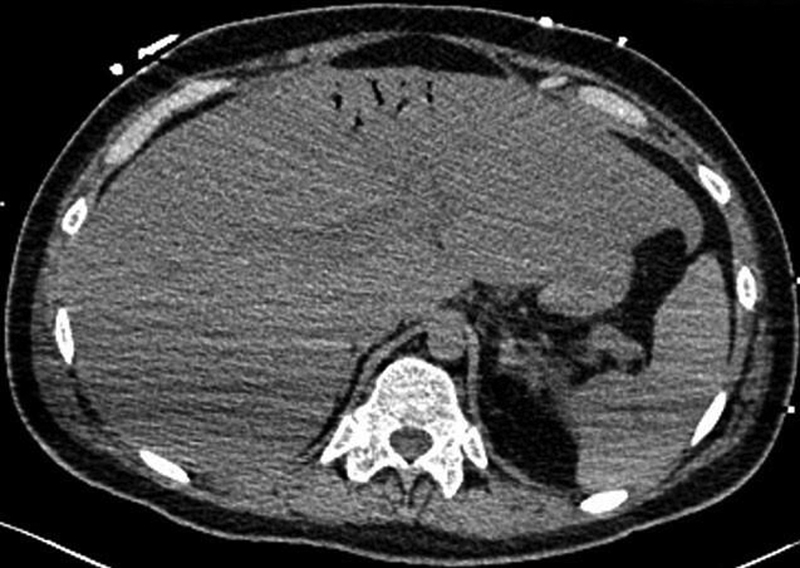
Axial noncontrast computed tomography (CT) scan image showing hepatic portal venous gas in the left portal vein branches.

Investigations revealed leukopenia and neutropenia (absolute neutrophil count of 20), elevated lactate level of 4.96 mmol/L, elevated C-reactive protein of 255 mg/L, elevated creatinine of 2.25 mg/dL (0.5–0.9), and normal procalcitonin. Stool test for clostridium difficile was negative. Plain CT scan showed HPVG, air in the superior mesenteric and ileocolic veins, and no pneumatosis intestinalis (PI), collection, or free air ( Figs. 1 2 3 ). The coronal CT image is schematically shown in Fig. 4 to aid the identification of the vessel branches.

**Fig. 2:**
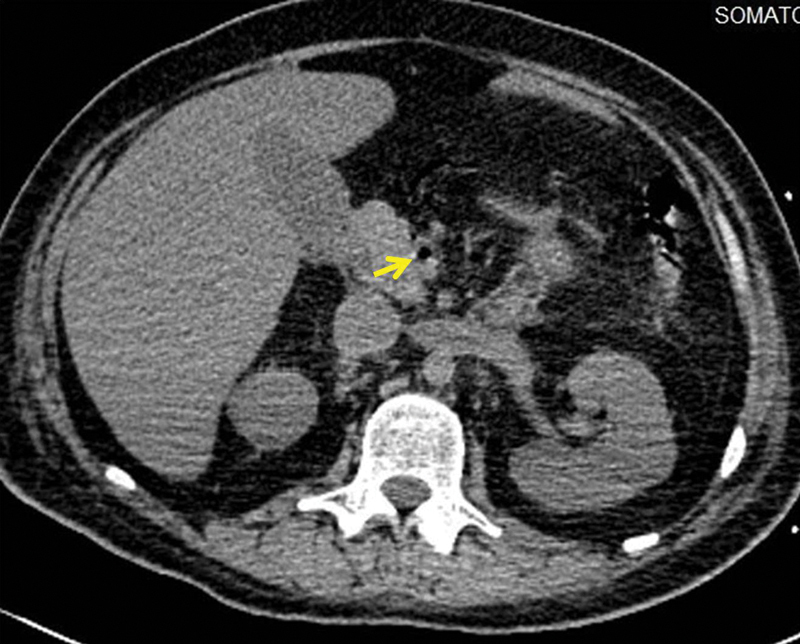
Axial noncontrast computed tomography (CT) scan image showing gas in the superior mesenteric vein ( *yellow arrow* ).

**Fig. 3:**
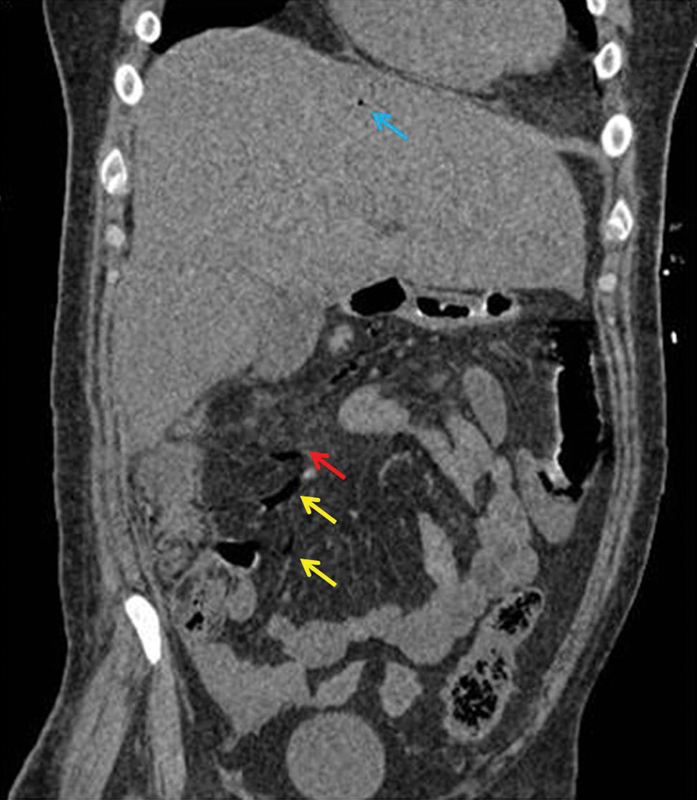
Coronal noncontrast computed tomography (CT) scan image showing air in the ileocolic vein ( *yellow arrow* ) and the right colic vein ( *red arrow* ), and hepatic portal venous gas ( *blue arrow* ). Also, air in the small tributaries of the superior mesenteric vein can be seen.

**Fig. 4:**
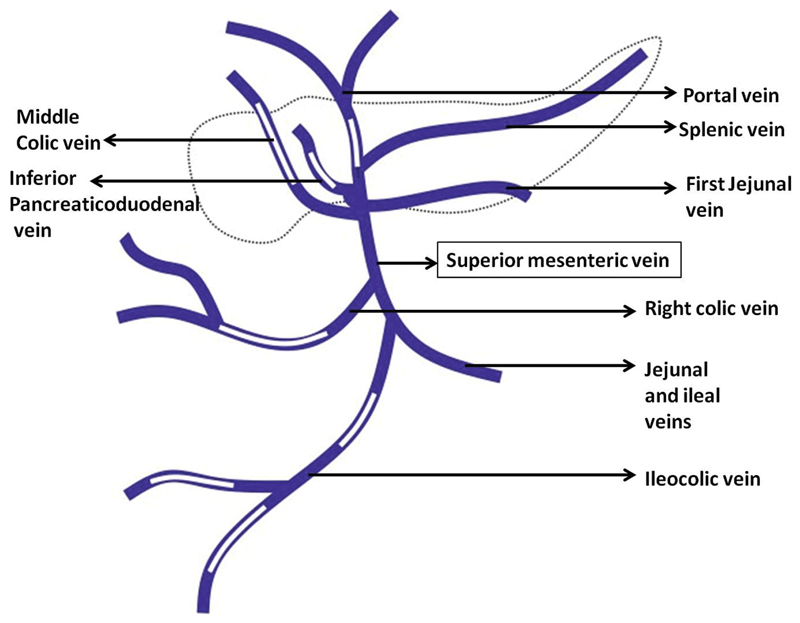
Schematic representation of the superior mesenteric vein branches corresponding to the coronal plane computed tomography (CT) scan with white filling defects in the diagram showing the location of portal vein air.

Bowel gangrene due to necrotizing enterocolitis, neutropenic colitis, pseudomembranous colitis, or ischemic colitis was considered in the differential diagnosis. However, abdominal examination did not reveal guarding or rigidity, and rebound tenderness was absent. There was no sudden clinical deterioration in the patient's condition. Clostridium difficile toxin was negative. There was no rise in procalcitonin, no elevation in lactate levels, or base excess on arterial blood gas analysis. There was no PI on CT scan. The patient was at a prohibitive risk of intervention due to his comorbid conditions, which included neutropenia, acute kidney injury, septic myocarditis, and tumor lysis syndrome. Hence, colonoscopy was not performed. A provisional diagnosis of neutropenic colitis was made for the patient, with steroid use as the probable cause of HVPG.

He was managed conservatively, with a working diagnosis of neutropenic enterocolitis as the possible cause for HPVG. A repeat CT with contrast after 15 days showed resolution of HPVG. He was discharged 21 days later. He is on salvage therapy for ALL with consideration for bone marrow transplant.

## Discussion

**Fig. 5:**
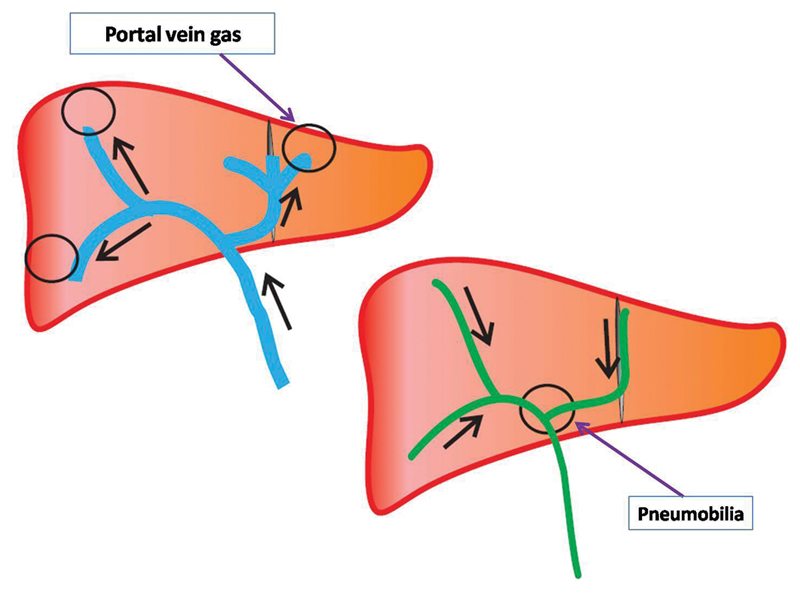
Location of pneumobilia and portal vein gas in the liver based on the direction of flow of bile and blood in the portal vein, respectively. Therefore, the portal vein gas is peripherally seen, within 2 cm of the liver capsule, and is usually multiple. On the other hand, pneumobilia is seen closer to the hepatic hilum.

HPVG occurs when intraluminal gas from overdistended intestine or infection with gas-producing bacteria enters the portomesenteric venous circulation. 4 Predisposing factors are intestinal mucosal damage, bowel distention, and sepsis. Intestinal obstruction, intestinal ischemia, inflammatory bowel disease, gastrointestinal neoplasms, and colonoscopy can damage the intestinal mucosa, which provides a portal for intraluminal gas to enter the intestinal wall and eventually portal venous system. However, HPVG has been associated with intra-abdominal abscess without mucosal damage in some cases, wherein the gas is produced by a gas-forming organism. 7 On imaging, HPVG can be differentiated from pneumobilia by the typical location as explained by the direction of flow of fluids in both the systems containing air, as shown in Fig. 5 . 4 6 7

**Fig. 6:**
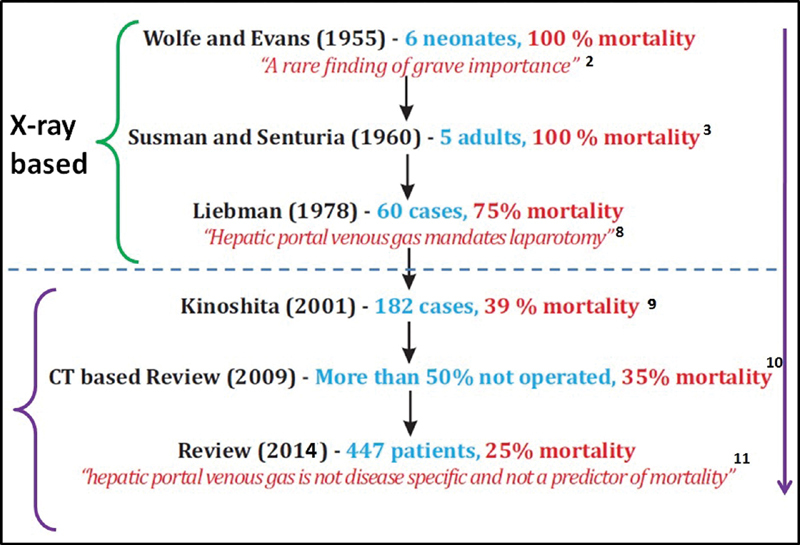
Mortality trends in patients with hepatic portal venous gas in the literature.

Until 1975, HPVG was considered synonymous with bowel gangrene with 100% mortality. 4 6 However, studies in recent times showed a drastic drop in mortality to 25 to 39%. 5 6 7 This decline in mortality was a result of recognition of diverse causes of HPVG by increased use of CT scans. These mortality trends are shown in Fig. 6 . 2 3 8 9 10 11

However, not all causes of HPVG produce the same outcomes. he presence of HPVG with PI indicates a high likelihood of bowel gangrene. 12 PI is gas in the bowel wall and is associated with HPVG in 50% of cases. When PI is present with HPVG, there is 49% mortality, and without PI, mortality is 29%. 6 13 PI is traditionally considered a sign of bowel wall infarction and a surgical emergency, especially when associated with HPVG. However, numerous nonischemic causes of PI have been described, such as inflammatory bowel disease, intestinal dilatation, connective tissue disease, organ transplantation or post-operative change, immune-deficiency status, and chemotherapy. 5 6 7 12

In modern times, HPVG has been recognized most commonly to arise due to iatrogenic causes such as upper and lower gastroinetstinal endoscopy, enemas, cardiac catheterization, vascular interventions, and interventional radiological procedures. 5 6 10 HPVG induced by these causes show a quiescent clinical course and require conservative management. Respiratory causes such as emphysema, cystic fibrosis, pancreatitis, hepatitis, seizure disorders, and intestinal pseudo-obstruction can also produce HPVG. The mortality rates reported for these causes range between 0 and 30%, which is way below that for cases where HPVG and PI occur due to bowel gangrene (75–80%). 11

The management of patients with radiological finding of HPVG thus varies from surgical intervention to completely conservative management, as seen in our case. 14 15 The key is to segregate patients into three groups based on a possible cause of HPVG. The first group of patients with mechanical intestinal pathology or bowel gangrene require emergent laparotomy. The second group of patients with probable mesenteric ischemia will benefit from a diagnostic laparoscopy. The third group of patients with other causes as described previously will need just conservative management. Thus, an evaluation for the specific cause of PI is clinically important to avoid unnecessary surgery, leading to improved clinical outcomes of the patients. Wayne et al established a vascular disease score for HPVG based on risk factor evaluation for thromboembolism, patient examination, lactate levels, and radiological finding of PI, which could help to determine the presence of mesenteric ischemia. 14 15

Patients with PI and clinical evidence of peritonitis or bowel perforation, old age, postoperative patient, recent vascular graft, surgery, hypercoagulable state, uncontrolled diabetes, and hypertension need careful evaluation to rule out bowel ischemia. 15 16 Worsening leukocytosis, increasing acidosis, and elevated lactates are red flag signs of bowel ischemia and/or gangrene. Radiological findings showing gas under the diaphragm, mechanical obstruction/strangulation, intra-abdominal abscess, and PI are indicators of need for surgery. If none of these factors are present, close monitoring is enough for other causes of HPVG. 16 17 Our case showed characteristics of HPVG, with the probable causes being chemotherapy and steroids after ruling out all other causes, and the patient was managed conservatively with a good outcome.

## Conclusion

HVPG is a radiological finding and is not synonymous with bowel gangrene and, hence, is not always an indication for surgery. However, HVPG coexisting with PI in a patient portends a poor prognosis and suggests bowel ischemia due to varied causes. Thus, the management of patients with radiological finding of HPVG varies from emergent surgical intervention to completely conservative management based on its etiology. The segregation of patients into etiological groups of mechanical cause with/without bowel gangrene, probable bowel ischemia, and nonischemic benign causes is practical and helpful in the patient's management when HVPG is present.
